# Advanced Endoscopic Imaging

**DOI:** 10.1155/2013/206839

**Published:** 2013-04-04

**Authors:** Helmut Neumann, Klaus Mönkemüller, Markus F. Neurath, Arthur Hoffman, Charles Melbern Wilcox

**Affiliations:** ^1^Department of Medicine I, University of Erlangen-Nuremberg, Ulmenweg 18, 91054 Erlangen, Germany; ^2^Basil Hirschowitz Endoscopic Center of Excellence, University of Alabama, Birmingham, AL, USA; ^3^St. Marienkrankenhaus Katharina-Kasper, Richard-Wagner Straße 14, 60318 Frankfurt am Main, Germany

It was in the late 18th century when the American essayist, poet, and philosopher Henry David Thoreau quoted “*It's not what you look at that matters, it's what you see*.” Indeed, more than 200 years later, this phrase is still valid and relevant, especially in the field of gastrointestinal (GI) endoscopy.

Endoscopists in the whole world are working hard to improve diagnosis and therapy of our patients. Despite these efforts, we are still confronted with some limitations of GI endoscopy including the lack of detection of colon polyps (i.e., significant adenoma miss rates), delayed diagnosis, and difficult areas to access, like the pancreatobiliary tract or the small bowel.

In the attempt to overcome these limitations, new endoscopic techniques are constantly being introduced. New endoscopic imaging techniques now allow for a more detailed analysis of mucosal and submucosal structures and include virtual chromoendoscopy, magnification endoscopy, and endocytoscopy. Various studies have shown the usefulness of these imaging techniques for conditions such as Barrett's esophagus, colon polyps, and early neoplasias of the luminal GI tract. Moreover, the recently introduced confocal laser endomicroscopy (CLE) system allows us to analyze structures at the cellular and subcellular layer thereby obtaining an optical biopsy during ongoing endoscopy. Besides, CLE has the potential to visualize fluorescence labeled structures against specific epitopes, that is, in gastrointestinal cancer or inflammatory bowel disease, thus adding molecular imaging to the field of endoscopic research. Furthermore, with the development of balloon-assisted endoscopy and capsule endoscopy, the endoscopist is now able to visualize the entire small bowel. Lastly, visualization beyond the mucosa is also important. This is accomplished with endoscopic ultrasonography (EUS). EUS plays now a pivotal role for the management and therapy of various diseases. Through EUS, the “eye” of the endoscopist is extended beyond the lumen allowing for a detailed examination of most adjacent structures to the luminal GI tract.

This special issue focuses on the exiting new developments of GI endoscopy. We are proud to present original articles and state-of-the-art reviews on the latest developments in the field of advanced endoscopic imaging. We are aware that it is impossible to cover the entire spectrum of advanced endoscopy in only one issue. The presented topics, however, highlight some of the most current aspects, controversies, and recommendations in selected areas of advanced GI imaging.

B. E. Bluen and coworkers analyzed the impact of EUS-FNA on patient management. Files from 268 patients were evaluated. In the conclusion, the authors suggest that the diagnostic accuracy of EUS-FNA might be improved further by taking more FNA passes from suspected lesions, optimizing needle selection, having an experienced echo-endoscopist available during the learning curve, and lastly having a cytologist present during the procedure. C. Xu et al. reviewed the technique and applications of contrast-enhanced harmonic EUS (CH-EUS) in pancreatic diseases and compared the technique to computed tomography, magnetic resonance imaging, and conventional EUS. The authors concluded that CH-EUS could be used for adequate sampling of pancreatic tumors but could not replace EUS-FNA now. Mohammad-Alizadeh and coworkers evaluated the efficacy of limited sphincterotomy plus large balloon dilation for removal of biliary stones in a prospective nonrandomized study including 50 patients. The authors described that the combined approach is an effective and safe treatment in patients with challenging bile duct stones.

M. Iwatate et al. evaluated NBI (Olympus, Tokyo, Japan) and NBI combined with magnification for the evaluation of colorectal lesions and additionally gave an outlook on future prospects. Digital chromoendoscopy techniques were evaluated in two different trials of this special issue. C. E. O. dos Santos and coworkers presented the results of a prospective randomized study comparing the accuracy of Fujinon Intelligent Color Enhancement (FICE; Fujifilm, Tokyo, Japan) and real-time chromoendoscopy for the differential diagnosis of diminutive (<5 mm) neoplastic and nonneoplastic colorectal lesions and found that both approaches showed high accuracy for the histopathological diagnosis of diminutive colorectal lesions. S. Hancock et al. described the potential of i-scan (Pentax, Tokyo, Japan) in clinical endoscopic practice in upper and lower endoscopic procedures and described the potential usefulness of this technique for real-time diagnosis and characterization of GI lesions. The small bowel was discussed in two manuscripts of this special issue. H. Suzuki et al. described the successful treatment of early stage jejunal adenocarcinoma by endoscopic mucosal resection using double-balloon enteroscopy. Whereas there are several reports on polypectomy of the small bowel, this is the first report of EMR of this part of the GI tract. J. Chen and J. Lee reviewed the current development of machine-vision-based analysis of wireless capsule endoscopy, thereby focusing on the research that identifies specific gastrointestinal pathology and methods of shot boundary detection. CLE is an advanced endoscopic imaging technique. R. Pittayanon et al. described for the first time the learning curve of CLE for assessment of gastric intestinal metaplasia. The authors found that after a short session of training, even beginners could achieve a high level of accuracy with a substantial level of interobserver agreement. Foersch et al. described the potential of molecular imaging using CLE for imaging of COX-2 activity in murine sporadic and colitis-associated colorectal cancer. The authors pose a proof of concept and suggested the use of CLE for the detection of COX-2 expression during colorectal cancer surveillance. Finally, S. Peter et al. presented a state-of-the-art review on the potential of CLE for real-time histopathological evaluation of the pancreaticobiliary system. They concluded that the novel use of this technique is particularly of significance in differentiating indeterminate biliary strictures as treatment depends on an accurate and prompt diagnosis.

We highly appreciate the excellent contributions of all authors of this special issue and want to encourage all readers to be receptive on the rapidly growing field of advanced endoscopic imaging. Nonetheless, just utilizing a new method is not enough; a clear understanding of how the method is used and how the findings are interpreted is the key for a successful endoscopy: “*It's not what you look at that matters, it's what you see*.”

*Helmut Neumann* *Helmut Neumann* *Klaus Mönkemüller* *Klaus Mönkemüller* *Markus F. Neurath* *Markus F. Neurath* *Arthur Hoffman* *Arthur Hoffman* *Charles Melbern Wilcox* *Charles Melbern Wilcox*

